# Fibre‐Enriched High‐Carbohydrate (FEHC) Diet Modulates Inflammation Without Affecting Bone Health in Older Women With Obesity: A Randomised Clinical Trial

**DOI:** 10.1002/dmrr.70089

**Published:** 2025-09-30

**Authors:** Francesca Cannata, Viola Viola, Giulia Leanza, Alice Laudisio, Malak Faraj, Flavia Tramontana, Alessandra Piccoli, Rocky Strollo, Mauro Maccarrone, Fabrizio Russo, Gianluca Vadalà, Veronica Sansoni, Giovanni Lombardi, Giuseppe Banfi, Chiara Verdelli, Rocco Papalia, Nicola Napoli

**Affiliations:** ^1^ Department of Medicine and Surgery Research Unit of Endocrinology and Diabetes Università Campus Bio‐Medico di Roma Rome Italy; ^2^ Operative Research Unit of Osteo‐Metabolic and Thyroid Diseases Fondazione Policlinico Universitario Campus Bio‐Medico Rome Italy; ^3^ Division of Endocrinology Diabetes and Metabolism Baylor College of Medicine Houston Texas USA; ^4^ Operative Research Unit of Orthopedic and Trauma Surgery Fondazione Policlinico Universitario Campus Bio‐Medico Rome Italy; ^5^ Department of Human Sciences and Promotion of the Quality of Life San Raffaele Roma Open University Rome Italy; ^6^ European Center for Brain Research (CERC)/Santa Lucia Foundation Rome Italy; ^7^ Department of Clinical Applied Sciences and Biotechnology University of L’Aquila L'Aquila Italy; ^8^ Laboratory of Experimental Biochemistry and Advanced Diagnostics IRCCS Ospedale Galeazzi‐Sant’Ambrogio Milano Italy; ^9^ Departament of Athletics Strength and Conditioning Poznań University of Physical Education Poznań Poland; ^10^ Vita‐Salute San Raffaele University Milano Italy; ^11^ Division of Bone and Mineral Diseases Washington University in St. Louis St. Louis Missouri USA

**Keywords:** bone health, fibre‐enriched diet, inflammation, obesity, Wnt signalling

## Abstract

**Background:**

In older adults, obesity is associated with frailty, conditions worsened by age related decline in bone mineral density (BMD) and muscle mass.

**Objective:**

To evaluate whether a 3‐month Fibre‐Enriched High Carbohydrate (FEHC) diet preserves bone health, reduces inflammation and modulates Wnt signalling in older adults with obesity.

**Methods:**

In this clinical trial, 86 women aged 65–85 years with obesity (BMI ≥ 30 kg/m^2^) undergoing hip arthroplasty were assigned to a free control diet (FCD) or a FEHC diet (FEHC) group for 3 months before surgery. Clinical, systemic, and molecular assessments were performed, including gene expression analyses in bone and muscle tissues.

**Results:**

A significant reduction in waist circumference was observed over time in the FEHC group (*p* = 0.037), whereas no changes were detected in body weight, BMD or bone microarchitecture. Compared to FCD, the FEHC group showed reduced circulating IL‐6 (*p* = 0.03), IL‐8 (*p* = 0.022) and TNFα (*p* = 0.04) levels, along with lower IL‐6 gene expression in muscle (*p* = 0.035). A strong trend of increased IGF‐1 gene expression in muscle tissue of the FEHC group was also observed (*p* = 0.058). Gene expression analyses revealed a significant increase in WNT5a expression in muscle (*p* = 0.049), and an upward trend in WNT10b expression in bone (*p* = 0.055) while serum levels of DKK‐1 were significantly higher in the FEHC group compared to FCD (*p* < 0.0001).

**Conclusion:**

The FEHC diet reduces systemic and local inflammation, without affecting skeletal health in older adults with obesity.

## 1 Introduction

The global prevalence of obesity among older adults has risen significantly, representing a growing public health concern. This demographic shift is particularly alarming as the interplay between obesity and ageing exacerbates age‐related declines in physical function, leading to sarcopenia and osteopenia [1, 2, 3, 4]. These conditions are further amplified by visceral adiposity, a hallmark of obesity, which drives chronic low‐grade inflammation and metabolic dysfunction [5]. While this inflammatory cascade is a well‐known contributor to chronic diseases [6], its impact on skeletal health is more complex and not fully understood. Paradoxically, despite obesity being associated with an elevated bone mineral density (BMD), this protective effect does not translate into reduced fracture risk [7]. Instead, older adults with obesity exhibit a significant risk of fractures [8], underscoring the complex relationship between obesity and bone health. Central to these processes is the Wnt signalling, which plays a pivotal role in regulating bone remodelling and the inflammatory state [9, 10]. Wnt signalling promotes osteoblastogenesis and myogenesis while inhibiting adipogenesis [11]. However, advancing age is associated with a downregulation of Wnt signalling in animal models, a condition also observed in individuals with metabolic diseases [12, 13, 14], shifting cellular pathways towards adipogenesis and a pro‐inflammatory state [15]. Managing obesity in this population, however, remains a clinical challenge, as conventional interventions are frequently associated with adverse effects on bone and muscle health [16, 17]. Lifestyle therapies, including dietary interventions, are cornerstones of obesity treatment [18, 19]. However, their impact on age‐related inflammatory, and skeletal outcomes requires further investigation. Various dietary regimens have been proposed, with fibre‐enriched, high‐carbohydrate (FEHC) diets gaining attention for their metabolic and anti‐inflammatory benefits [20, 21]. Recent studies have demonstrated that FEHC diets significantly improve metabolic outcomes and modulate inflammatory parameters compared with standard dietary approaches [22]. Observational data have linked high fibre intake to a reduced risk of obesity [23, 24], while large cohort studies have shown associations between FEHC diets, higher plasma levels of adiponectin and lower C‐reactive protein (CRP) concentrations [25, 26]. This dietary pattern, rich in phenolic compounds, has been shown to decrease tumour necrosis factor‐alpha (TNF‐α) and interleukin‐6 (IL‐6) levels, suppress CRP and improve endothelial function [26, 27, 28]. Building on this, our group previously demonstrated that a 12‐week high‐fibre diet improved metabolic outcomes, including reductions in BMI and HbA1c, in patients with type 2 diabetes mellitus (T2DM) [29]. However, this intervention was associated with a reduction in bone formation markers without changes in bone microarchitecture, which is frequently observed in individuals with metabolic impairment [30], or Wnt signalling and inflammation‐related gene expression in bone tissue [29]. These findings emphasise the need for dietary interventions that address the challenges of obesity while preserving skeletal health. In light of these findings, the primary objective of this study was to assess whether a 3‐month FEHC diet preserves skeletal health, reduces obesity‐related inflammation, and modulates the Wnt signalling in older adults with obesity.

## 2 Methods

### 2.1 Participants

The Fibre‐Enriched High Carbohydrate (FEHC) diet trial in older women with obesity (CO‐2016‐02362346) was a 3‐arm randomised controlled trial conducted from September 2019 through January 2022 at the Divisions of Endocrinology and Orthopaedic Surgery at Policlinico Universitario Campus Bio‐Medico di Roma, Rome, Italy. The study received approval from the Ethics Committee of the Campus Bio‐Medico University of Rome, and all procedures were conducted in compliance with the Declaration of Helsinki. Written informed consent was obtained from all participants. Patients with osteoarthritis scheduled for hip arthroplasty were screened for participation. Eligibility criteria included: elderly (65–85 years old) women with obesity (BMI > 30 kg/m^2^) planned for hip replacement surgery based on the orthopaedic surgeon’s recommendation, ambulatory individuals willing and able to provide informed consent, and no current use of medications or presence of diseases affecting bone metabolism. Participants with any bone disease (such as osteoporosis, osteogenesis imperfecta, fibrous dysplasia or malignancy), calcium disorders, or hepatic or renal disease were excluded. In addition, participants were excluded if they were on any medical treatment that affected bone metabolism (e.g. teriparatide, romosozumab, raloxifene, bisphosphonates, denosumab, thiazolidinediones, glucocorticoids and anabolic steroids) or if they were current smokers.

### 2.2 Study Design and Randomisation

**FIGURE 1:**
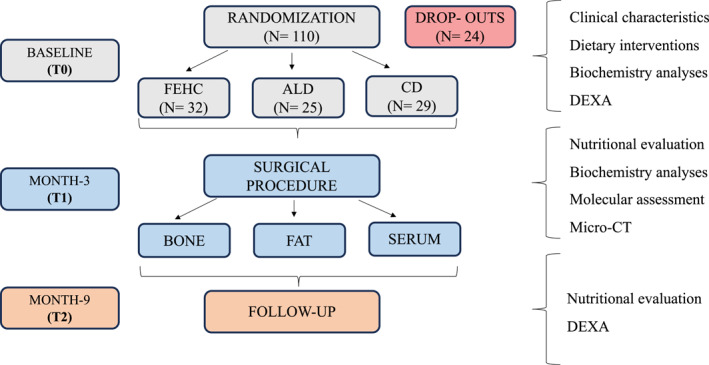
Schematic overview of the study design and randomisation. A total of one hundred and ten older women with obesity were randomised into three dietary intervention groups: Fibre‐enriched high‐carbohydrate diet (FEHC), ad libitum diet (ALD), and control diet (CD) (T0). Eighty‐six participants followed a dietary regimen for 12 weeks prior to hip arthroplasty (T1). After surgery, those in the FEHC group transitioned to a less restrictive isocaloric diet, which was continued for an additional 6 months (T2).

At the baseline visit (T0), participants were randomly assigned to one of three groups: (1) ad libitum diet group (ALD), (2) Fibre‐Enriched High Carbohydrate (FEHC) diet group or (3) a control diet group (CD). Participants adhered to their assigned dietary regimen for 12 weeks prior to undergoing hip arthroplasty (T1). Following the surgical procedure, participants in the FEHC group transitioned to a less restrictive isocaloric diet, which was maintained for an additional 6 months (T2). The randomisation sequence was computer generated, with stratification to ensure balance across BMI categories. Further details regarding the randomisation process and study design are provided in Figure 1.

### 2.3 Dietaries Intervention

Participants were prescribed one of three dietary regimens in accordance with the randomisation protocol. After randomisation, qualified personnel provided participants with a tutorial session to familiarise them with the dietary protocol and study requirements. Participants in the ALD group received general information about maintaining a healthy diet. For the FEHC and CD groups, energy intake was individualised based on each participant's estimated total daily energy expenditure, calculated using the Harris–Benedict equation (with sex‐specific formulas for men and women) and adjusted for physical activity level. These calculations were used to establish a caloric deficit aimed at promoting weight loss. A 24‐h dietary recall was also performed to assess habitual intake and better tailor the dietary recommendations. The FEHC diet was composed of whole grains, legumes, fermented products, and seaweeds, with no inclusion of animal products or added sugars. Daily energy intake was distributed as approximately 72% carbohydrates, 18% fat and 10% protein, with a fibre intake of 40 g per 1000 kcal. The FEHC diet was designed as a plant‐based, high‐carbohydrate, high‐fibre diet for the pre‐surgical phase, based on evidence generated by our research group demonstrating its metabolic and anti‐inflammatory benefits in individuals with obesity and metabolic syndrome [20, 21, 31]. Following the surgical procedure, the FEHC diet was modified to include fish‐derived proteins and vegetable oils to support nutritional needs during recovery, while maintaining its high carbohydrate and fibre content (70% carbohydrates, 17% fat, 13% protein; fibre intake: 38 g per 1000 kcal). This less restrictive isocaloric version of the diet was designed to ensure long‐term adherence while preserving its metabolic and anti‐inflammatory benefits [32]. The control diet adhered to the recommendations of the Italian Society of Obesity, with daily energy intake consisting of 45%–60% carbohydrates, 35% fat and 10%–20% protein. The recommended fibre intake was 20 g per 1000 kcal per day. All participants were supplemented to ensure an intake of 1000 mg of calcium and 1000 IU of vitamin D per day. The goal was to achieve a weight loss of approximately 10% of their baseline body weight within 3 months and to maintain it for an additional 6 months. Dietary compliance for all the participants was monitored through monthly follow‐up visits with the study dietitian and weekly telephone check‐ins.

### 2.4 Anthropometric and Biochemical Assessments

Body weight was measured at each time point (T0, T1 and T2) using a standard weighing scale, and BMI was calculated as weight in kilograms (kg) divided by height in metres squared (kg/m^2^). Fasting morning blood samples were collected at each time point for the analysis of glucose and HbA1c using standard laboratory methods. Total cholesterol and triglycerides (TG) were quantified using automated enzymatic commercial kits (Miles/Technicon, Tarrytown, NY), while HDL‐c was measured after the precipitation of apolipoprotein‐B‐containing lipoproteins using dextran sulphate and magnesium [33]. LDL‐c levels were calculated using the Friedewald equation [34].

### 2.5 Bone Assessment

#### 2.5.1 Dual Energy X‐Ray Absorptiometry (DXA)

Lumbar spine (L1–L4), and total femoral BMD were measured by DXA (QDR‐4500A; Hologic Inc., Bedford, MA, USA) at the lumbar spine and proximal femur. The T‐scores for both sites were recorded, and the coefficient of variation (CV) for this technique at our centre was 1.1% for the lumbar spine and 1.2% for the proximal femur. All scans were performed and analysed using standardised procedures [35].

#### 2.5.2 Micro‐Computed Tomography (μCT) Analysis

The bone mass and microstructure of the inferomedial femoral neck were analysed using micro‐computed tomography (μCT 40, Scanco Medical AG, Switzerland). Bone cores were placed in a sample holder and fixed with sponges to prevent movement during scanning. The scans were performed using standardised settings of 55 kV, 145 μA and 200 ms integration time, producing image stacks with an isotropic voxel size of 15 μm. For analysis, the cortical and trabecular bone regions of interest were manually contoured, excluding the transitional zone and damaged border regions. Trabecular bone was binarised using a global threshold 450 mg HA/cm^3^ to calculate total volumetric bone mineral density (vBMD, mg/cm^3^), cross‐sectional area (mm^2^), trabecular vBMD (mg/cm^3^), trabecular area (mm^2^), trabecular number (mm^−1^) and trabecular thickness (mm). For the cortical bone, a global threshold of 550 mg HA/cm^3^ was used to assess cortical vBMD, (mg/cm^3^), cortical area (mm^2^), cortical thickness (mm) and cortical porosity (%). Analyses were conducted using XamFlow software (Lucid Concept AG, Zurich, Switzerland). Measurements of thickness and spacing were based on a model‐independent, volume‐based assessment method, as described by Hildebrand and Rüegsegger [36].

#### 2.5.3 Systemic Inflammation and Bone Metabolic Markers

Serum samples were analysed for circulating inflammatory and metabolic mediators using a multiplex assay (Biotechne) based on Luminex XMAP technology (MAGPIX, BioRad). Frozen serum samples were thawed and centrifuged at 16,000 × g for 4 min before analysis. Adiponectin was measured using a single‐analyte panel (Luminex Human Discovery Assay, 1‐Plex), and cytokines, including IL‐6, IL‐8, IL‐10, IL‐15, TNFα, MCP1, DKK1 and sclerostin, were measured using high‐sensitivity and discovery panels (Luminex Performance Human High Sensitivity Cytokine Magnetic Panel A and Luminex Human Discovery Assay). All assays were conducted in duplicate. Samples were diluted according to assay requirements: a 300‐fold dilution for the adiponectin panel and a 2‐fold dilution for multiplex panels. Standards, controls, and diluted samples (100 μL for high‐sensitivity assays and 50 μL for discovery assays) were added to wells containing 25 μL of magnetic microparticle cocktail pre‐coated with analyte‐specific antibodies. Plates were incubated overnight at 4°C on a horizontal orbital microplate shaker set at 800 rpm. After three washes with the assay wash buffer, 50 μL of biotin‐labelled detection antibodies were added to each well and incubated for 1 h at room temperature with gentle shaking. Following another set of washes, 50 μL of Streptavidin‐PE solution was added and incubated for 30 min at room temperature. After a final set of washes, the microparticles were resuspended in 100 μL of wash buffer and analysed within 90 min using the Luminex MAGPIX analyser. The assay sensitivity was 0.31 pg/mL for IL‐6, 0.07 pg/mL for IL‐8, 0.24 pg/mL for IL‐10 and 0.54 pg/mL for TNFα. For MCP‐1, DKK1, and sclerostin, the sensitivity was 9.9, 50.9 and 7.0 pg/mL, respectively. The adiponectin sensitivity was 148 pg/mL.

### 2.6 Gene Expression in Bone and Muscle Tissue

#### 2.6.1 Sample Preparation

During the surgery, femoral head and muscle specimens from the vastus lateralis were collected for downstream analyses. Trabecular bone and muscle tissue were carefully transferred to sterile 50‐mL tubes containing sterile phosphate‐buffered saline (PBS) 1× and underwent repeated washing to remove contaminants. After each wash, the samples were vigorously vortexed, and the supernatant was discarded. This process was repeated at least twice to ensure purity. The following washing, bone and muscle tissue were snap frozen in liquid nitrogen and stored at −80°C until further analysis. Trabecular bone and muscle tissue samples, approximately 100 mg each, were used for RNA extraction. Additionally, bone cores (10–15 mm in diameter and 10–20 mm in length) were drilled from the inferomedial femoral neck, fixed in 4% paraformaldehyde for 3 days, and subsequently stored in PBS at 4°C until μCT analysis. To minimise contamination, all procedures were performed under sterile conditions.

#### 2.6.2 Isolation of mRNA and Evaluation of Gene Expression With RT‐PCR

Total RNA was isolated using TRIzol Reagent (Invitrogen Corp., Carlsbad, CA, USA) [37] following manufacturer's instructions, and RNA concentration was determined spectrophotometrically (InfiniteM200PRO; Tecan, Männedorf, Switzerland). The purified RNA was quantified using a NanoDrop spectrophotometer (Thermo Fisher Scientific) and considered suitable if the A260/A280 ratio was ∼2 and the A260/A230 ratio > 1.7. We only reverse‐transcribed RNA within this range, excluding from the analysis those samples not reaching this threshold. In order to carry out the quantitative real‐time RT‐PCR, 1 μg of total RNA was reverse transcribed using the High‐Capacity cDNA Reverse Transcription Kit (Applied Biosystems, Carlsbad, CA, USA), according to the product protocol (25°C for 10 min, 37°C for 2 h, 85°C for 5 min). A further real‐time RT‐PCR analysis was performed using the TaqMan gene expression analysis assay, with the 7900 real‐time PCR system (Applied Biosystems) and standard cycling conditions (95°C for 10 min; 40 cycles of 95°C for 15 s and 60°C for 1 min; followed by 95°C for 15 s, 60°C for 15 s, and 95°C for 15 s). Gene expression of WNT5A (Hs00998537_m1), WNT10B (Hs00928823_m1), SOST (Hs00228830_m1), IGF‐1 (Hs01547656_m1), IL‐6 (Hs00174131_m1), IL‐8 (Hs00174103_m1), IL‐10 (Hs00961622_m1), TNFα (Hs00174128_m1), ADIPOQ (Hs00605917_m1), OCN (Hs01587814_g1), RUNX2 (Hs00231692_m1), DKK‐1 (Hs00183740_m1), LEF‐1 (Hs01547250_m1), COL1A1 (Hs00164004_m1) were evaluated in bone and muscle tissue biopsies. Gene expression was normalised with Beta‐actin (Hs01060665_g1) for bone tissue, and GAPDH (Hs02786624_g1) for muscle tissue. The results were also normalised using the BestKeeper gene, which represents the adjusted mean of the three housekeeping genes for each sample [38, 39].

#### 2.6.3 Statistical Analyses

Statistical analyses were performed using the statistical package for social science (SPSS) for Mac 26.0. Differences were considered significant at the *p* < 0.050 level. Normality was assessed using the Kolmogorov–Smirnov test, whereas homogeneity of variances was evaluated with Levene's test. Data of continuous variables are presented as mean ± standard deviation (SD), and non‐normally distributed variables are reported as medians with interquartile ranges (IQR). For clinical parameters, such as bone assessment, the three original diet groups were maintained. Between‐group comparisons were conducted using one‐way analysis of variance (ANOVA) for normally distributed variables. Within‐group comparisons across time points (T0, T1, T2) were assessed using the Friedman test, followed by post hoc Wilcoxon tests with Bonferroni's adjustment for multiple comparisons. For analyses of gene expression and systemic parameters, two (ALD and CD) of the original groups were combined based on the absence of significant differences in anthropometric and metabolic outcomes after the intervention. For these analyses, between‐group differences were evaluated using the Mann–Whitney *U* test or independent *t*‐test, depending on the data distribution. Body parameters were compared before and after the dietetic intervention by using the Wilcoxon signed‐rank test. The Friedman test followed by the Wilcoxon test with Bonferroni's adjustment was adopted to compare parameters of each diet group at T0, T1 and T2. Sample size estimation was based on available data from prior studies [40] and focused on outcomes including, TNF‐α mRNA expression, trabecular bone volume, and sclerostin levels. Calculations indicated a required sample size ranging from 7 to 23 participants per group, depending on the outcome. To ensure sufficient statistical power and account for potential dropouts, the study aimed to enrol approximately 30 participants per group.

## 3 Results

### 3.1 Baseline Characteristics

A total of 86 participants were enroled in this study, with baseline characteristics comparable across the three groups: ALD (*n* = 25), FEHC (*n* = 32) and CD (*n* = 29), as shown in Table 1. The mean age of participants was comparable. Body weight and body mass index (BMI) did not differ between the groups, as did waist circumference. Fasting glucose, HBA1c, total cholesterol levels, HDL, LDL and triglycerides serum levels were similar among the groups.

**TABLE 1:** Baseline clinical characteristics at T0.

Parameters	ALD (*n* = 25)	FEHC (*n* = 32)	CD (*n* = 29)	*p* value
Age (years)	73.36 ± 6.53	71.90 ± 5.59	72.41 ± 6.73	0.69
Weight (kg)	89.17 ± 13.47	86.80 ± 13.00	93.01 ± 13.93	0.20
BMI (kg/cm^2^)	33.47 ± 4.94	33.18 ± 3.93	35.15 ± 4.12	0.17
Waist circumference (cm)	103.6 ± 11.65	103.8 ± 10.18	103.6 ± 9.54	0.99
Fasting blood glucose (mg/dL)	107.1 ± 23.46	101.8 ± 16.07	105.8 ± 24.02	0.61
HbA1c (%)	6.28 ± 0.97	5.94 ± 0.76	6.53 ± 1.39	0.51
Total cholesterol (mg/dL)	202.4 ± 33.29	190.3 ± 38.90	205.1 ± 28.92	0.20
HDL (mg/dL)	43.96 ± 11.15	47.81 ± 18.56	44.14 ± 9.92	0.49
LDL (mg/dL)	135.90 ± 34.79	121.9 ± 37.65	138.5 ± 28.63	0.13
Triglycerides (mg/dL)	124.9 ± 41.17	123.8 ± 45.56	130.8 ± 35.36	0.81

*Note:* Data are presented as mean ± standard deviation.

### 3.2 Anthropometric and Metabolic Changes

**FIGURE 2:**
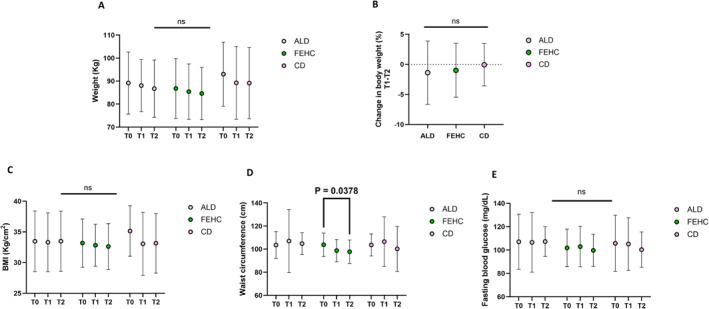
Anthropometric and metabolic parameters at baseline (T0), month‐3 (T1), and after month‐9 (T2) in ad libitum diet (ALD, gray circles), a fiber‐enriched high‐carbohydrate diet (FEHC, green circles), and a control diet (CD, purple circles) group. (A) Body weight (kg). (B) Change in body weight (%) between T1 and T2. (C) Body mass index (BMI, kg/m²). (D) Waist circumference (cm). (E) Fasting blood glucose (mg/dL). Data are presented as mean ± standard deviation (SD). Statistical analysis was performed using two‐way ANOVA followed by Tukey’s multiple comparisons test. Comparisons were considered statistically significant at *p* < 0.05; all others were not significant (ns).

Anthropometric parameters revealed no significant changes in body weight or BMI within or between groups at any timepoint, as shown in Figure 2A–C. However, the FEHC group exhibited a significant reduction in waist circumference from T0 to T2 (*p* = 0.0378), as shown in Figure 2D. No significant changes in fasting blood glucose levels were observed (Figure 2E).

### 3.3 Comprehensive Bone Assessment

#### 3.3.1 Bone Mineral Density

**FIGURE 3:**
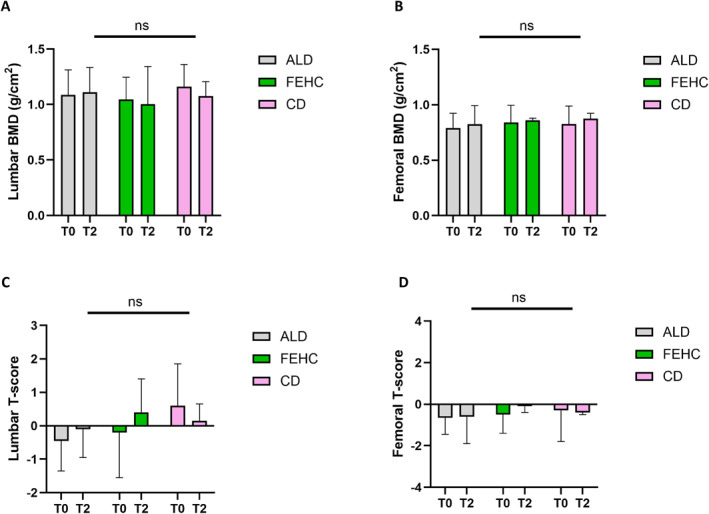
Analysis of Bone mineral density (BMD) and T‐scores by Dual‐energy X‐ray Absorptiometry (DXA) at baseline (T0) and after month‐9 (T2) in ad libitum diet (ALD, gray bars), a fiber‐enriched high‐carbohydrate diet (FEHC, green bars), and a control diet (CD, pink bars) group. (A) Lumbar BMD (g/cm²). (B) Femoral BMD (g/cm²). (C) Lumbar T‐score. (D) Femoral T‐score. Data are presented as mean ± standard deviation (SD). Statistical analysis was performed using two‐way ANOVA followed by Tukey’s multiple comparisons test. Comparisons were considered statistically significant at *p* < 0.05; no significant differences were observed (ns).

Bone mass parameters, including lumbar and femoral BMD, were evaluated at T0 and T2. No significant changes in lumbar (Figure 3A) and femoral BMD (Figure 3B) were detected within any experimental group over time. Similarly, comparisons between groups at both time points did not reveal any statistically significant differences. T‐score analyses for lumbar (Figure 3C) and femoral BMD (Figure 3D) confirmed these results, showing consistent values across all groups and time points.

#### 3.3.2 Bone Microarchitecture

High‐resolution microarchitectural analyses of trabecular and cortical bone parameters showed no significant differences among groups, as detailed in Table S1.

### 3.4 Circulating Serum Markers of Inflammation and Wnt Signalling

Inflammatory and Wnt signalling markers were analysed in serum samples collected at T0–T1 (Table 2). At T0–T1, levels of IL‐6 were significantly lower in the FEHC group compared to the FCD group (*p* = 0.030). IL‐8 levels were also significantly lower in the FEHC group compared to the FCD group (*p* = 0.022). IL‐10 levels were similar between the groups, as were IL‐15 levels. TNFα levels were significantly lower in the FEHC group compared to the FCD group (*p* = 0.040). DKK1 levels were higher in the FEHC group compared to the FCD group (*p* < 0.0001). No significant differences were observed for MCP‐1, Sclerostin or adiponectin.

**TABLE 2:** Serum levels of inflammatory and Wnt signalling markers in the FEHC and FCD groups from T0 to T1.

T0–T1	FEHC (*N* = 32) (median and IQR range)	FCD (*N* = 54) (median and IQR range)	*p* value
IL‐6 (pg/mL)	2.22 (1.41 to 3.07)	+3.04 (2.37 to 5.53)	**0.030**
IL‐8 (pg/mL)	4.00 (1.86 to 5.44)	+6.68 (5.23 to 9.29)	**0.022**
IL‐10 (pg/mL)	1.08 (0.56 to 1.18)	1.06 (0.98 to 1.31)	0.198
IL‐15 (pg/mL)	1.06 (0.37 to 3.97)	1.98 (1.69 to 3.34)	0.638
TNFα (pg/mL)	6.05 (3.94 to 7.06)	+7.52 (6.18 to 9.00)	**0.040**
MCP1 (pg/mL)	296.42 (253.85 to 337.97)	319.47 (284.64 to 363.74)	0.445
DKK1 (pg/mL)	7183.1 (5085.6 to 7907.7)	−3667.8 (2935.9 to 4888.4)	**<** **0.0001**
Sclerostin (pg/mL)	204.7 (150.4 to 334.5)	207.4 (184.4 to 255.4)	0.546
Adiponectin (pg/mL)	10,039 (8229 to 12,103)	10,050 (8253 to 11,051)	0.748

*Note:* Data are expressed as fold change values (T1/T0) and reported as medians with interquartile ranges (IQR). Positive and negative signs indicate the direction of change in comparison to the other group. Significant data are presented in bold.

### 3.5 Gene Expression of Wnt Signalling, Bone Metabolism and, Inflammation‐ Related Genes in Bone Tissue

**FIGURE 4:**
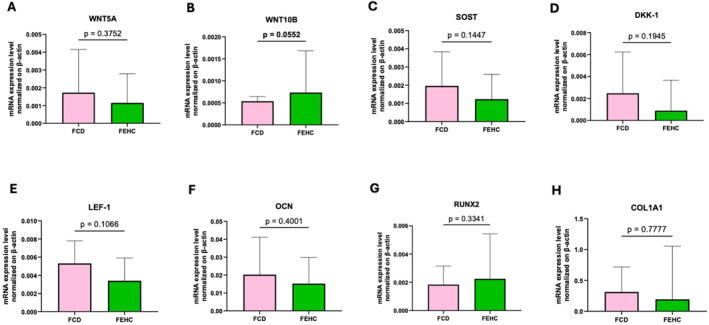
Gene expression analysis by Real‐time PCR of Wnt signaling and bone metabolism–related genes in bone tissue at T1 in a fiber‐enriched high‐carbohydrate diet (FEHC, green bars) and a control diet (CD, pink bars) group. (A) Wnt family member 5A (WNT5A). (B) Wnt family member 10B (WNT10B). (C) Sclerostin (SOST). (D) Dickkopf WNT signaling pathway inhibitor 1 (DKK1). (E) Lymphoid enhancer‐binding factor 1 (LEF1). (F) Osteocalcin (OCN). (G) Runt‐related transcription factor 2 (RUNX2). (H) Collagen type I alpha 1 chain (COL1A1). Data are presented as median values with interquartile ranges (IQR). Statistical analysis was performed using two‐way ANOVA followed by Tukey’s multiple comparisons test. Comparisons were considered statistically significant at *p* < 0.05.

**FIGURE 5:**
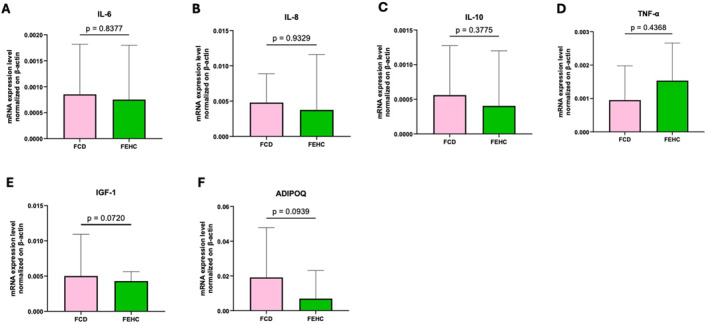
Gene expression analysis by Real‐time PCR of inflammatory and metabolism‐related genes in bone tissue at T1 in a fiber‐enriched high‐carbohydrate diet (FEHC, green bars) and a control diet (CD, pink bars) group. (A) Interleukin 6 (IL‐6). (B) Interleukin 8 (IL‐8). (C) Interleukin 10 (IL‐10). (D) Tumor necrosis factor alpha (TNF‐α). (E) Insulin‐like growth factor 1 (IGF‐1). (F) Adiponectin (ADIPOQ). Data are presented as median values with interquartile ranges (IQR). Statistical analysis was performed using two‐way ANOVA followed by Tukey’s multiple comparisons test. Comparisons were considered statistically significant at *p* < 0.05.

Gene expression analysis in bone tissue revealed a trend towards higher WNT10B expression in the FEHC group compared to the FCD group (*p* = 0.0552), as shown in Figure 4B. No other genes in the panel showed statistically significant differences between the groups (Figure 4A,C–H). Similarly, the expression of inflammation‐related genes did not significantly differ between the groups (Figure 5A–F).

### 3.6 Gene Expression of Wnt Signalling, Bone Metabolism, and Inflammation‐ Related Genes in Muscle Tissue

**FIGURE 6:**
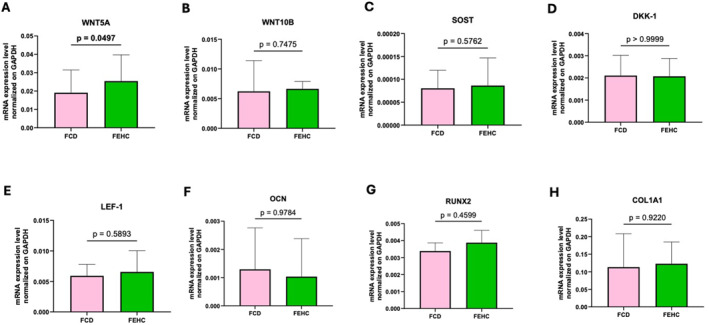
Gene expression analysis by Real‐time PCR of Wnt signaling and bone metabolism–related genes in muscle tissue at T1 in a fiber‐enriched high‐carbohydrate diet (FEHC, green bars) and a control diet (CD, pink bars) group. (A) Wnt family member 5A (WNT5A). (B) Wnt family member 10B (WNT10B). (C) Sclerostin (SOST). (D) Dickkopf WNT signaling pathway inhibitor 1 (DKK1). (E) Lymphoid enhancer‐binding factor 1 (LEF1). (F) Osteocalcin (OCN). (G) Runt‐related transcription factor 2 (RUNX2). (H) Collagen type I alpha 1 chain (COL1A1). Data are presented as median values with interquartile ranges (IQR). Statistical analysis was performed using two‐way ANOVA followed by Tukey’s multiple comparisons test. Comparisons were considered statistically significant at *p* < 0.05.

**FIGURE 7:**
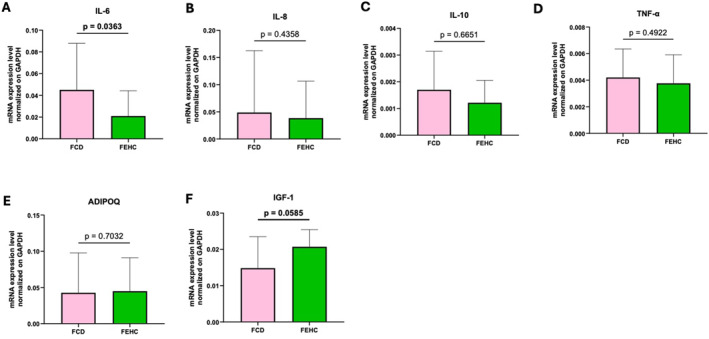
Gene expression analysis by Real‐time PCR of inflammatory and metabolism‐related genes in muscle tissue at T1 in a fiber‐enriched high‐carbohydrate diet (FEHC, green bars) and a control diet (CD, pink bars) group. (A) Interleukin 6 (IL‐6). (B) Interleukin 8 (IL‐8). (C) Interleukin 10 (IL‐10). (D) Tumor necrosis factor alpha (TNF‐α). (E) Insulin‐like growth factor 1 (IGF‐1). (F) Adiponectin (ADIPOQ). Data are presented as median values with interquartile ranges (IQR). Statistical analysis was performed using two‐way ANOVA followed by Tukey’s multiple comparisons test. Comparisons were considered statistically significant at *p* < 0.05.

Gene expression analysis in muscle tissue revealed a significantly higher expression of WNT5A in the FEHC group compared with the FCD group (*p* = 0.0497; Figure 6A). No other significant differences were observed in genes involved in Wnt signalling or bone metabolism (Figure 6B–H). Regarding inflammation‐related genes, IL‐6 expression was significantly lower in the FEHC group compared to the FCD group (*p* = 0.0363; Figure 7A), while IGF‐1 expression showed a positive trend in the FEHC group compared to the FCD group (*p* = 0.0585; Figure 7F). No other significant differences were detected among the remaining inflammatory genes (Figure 7B–E).

## 4 Discussion

Our data provide a comprehensive evaluation of the effects of a fibre‐ and carbohydrate‐rich diet on skeletal health in older adults with obesity. By integrating clinical, systemic, and molecular assessments, we examined the effect of the FEHC diet on modulating inflammation and, in turn, impacting bone health, addressing a critical challenge in managing frailty in this high‐risk population. Our findings demonstrated that the FEHC diet did not negatively impact bone health, including BMD and microarchitectural integrity. This is particularly relevant in older adults with obesity, a population at increased risk of musculoskeletal impairment due to ageing and metabolic burden [41]. While conventional dietary interventions often raise concerns about potential skeletal complications [19, 42, 43, 44, 45], our data suggest that the FEHC diet may be a safe nutritional strategy that supports metabolic improvements without compromising bone quality. In addition, the FEHC diet showed a significant reduction in waist circumference, a key marker of central adiposity [46]. This finding is particularly important because visceral fat is a major driver of chronic inflammation and metabolic dysfunction in older adults [47]. The reduction in central adiposity likely contributed to the improvements in functional outcomes we previously reported in the same cohort [48], as reflected by increased scores in the Oxford Hip Score (OHS), the Western Ontario and McMaster Universities Osteoarthritis Index (WOMAC) and the Hip Disability and Osteoarthritis Outcome Score (HOOS). In that study, patients following the FEHC diet experienced significant improvements in physical function and pain [48]. These functional gains suggest that alleviating central adiposity may reduce joint stress [49] and inflammation [50], improving mobility and quality of life. These outcomes suggest that the unique composition of the FEHC diet, rich in fibre and complex carbohydrates, may contribute to its role in modulating inflammation [44]. At a systemic level, the FEHC diet demonstrated a reduction in key pro‐inflammatory markers. These findings align with the study by Shivakoti et al., which investigated the relationship between dietary fibre intake and inflammatory markers in older adults [51]. In their study, higher fibre consumption, particularly from cereals, was significantly associated with lower levels of IL‐6 and C‐reactive protein (CRP) [51], both critical markers of systemic inflammation. Similarly, in our study, the FEHC diet significantly lowered IL‐6 and IL‐8 levels, confirming its anti‐inflammatory action. This finding is particularly relevant given the established role of IL‐6 and IL‐8 in promoting chronic inflammation and their association with musculoskeletal disorders such as osteoporosis and sarcopenia [52]. There is much evidence that dietary fibre might be associated with decreased inflammatory reactions [26, 27, 53, 54, 55]. Most of these effects appear to be mediated by the production of short‐chain fatty acids (SCFAs). SCFAs directly inhibit the NF‐κB signalling pathway, which regulates the transcription of genes encoding pro‐inflammatory cytokines [53]. Furthermore, the significant reduction in TNFα levels observed in the FEHC group is consistent with the mechanisms through which fibre‐rich diets are known to mitigate systemic inflammation. However, while the relationship between dietary fibre and systemic inflammation is well‐documented, its direct role in musculoskeletal tissues remains less explored. Therefore, we investigated the effect of the FEHC diet on molecular mechanisms in both bone and muscle tissues. In bone tissue, the expression of WNT10b, a key activator of osteoblast differentiation and bone formation [56], showed a strong trend towards an increase in the FEHC group. This observation is consistent with findings from animal models, highlighting the critical role of WNT10B in maintaining bone mass and supporting osteoblast activity. Stevens et al. reported that WNT10b deficiency leads to age‐related bone loss and a progressive reduction in mesenchymal progenitor cells [57]. Similarly, transgenic overexpression of WNT10b in mice has been shown to increase bone mass and strength [58], while its genetic ablation leads to reduced bone mass [59, 60], as well as a decreased number and impaired function of osteoblasts [59]. In contrast, circulating levels of DKK1 were higher in the FEHC group. As DKK1 is a potent inhibitor of the Wnt/β‐catenin signalling pathway, its elevated serum levels would typically suggest suppressed Wnt signalling and reduced osteogenic activity [61]. However, the observed trend in WNT10b expression may reflect a compensatory mechanism that helps preserve osteoblast function and bone remodelling despite systemic inhibition. While no significant changes in inflammatory gene expression were observed at the bone tissue level, the FEHC diet showed anti‐inflammatory effects in muscle tissue, as evidenced by significantly lower IL‐6 gene expression. IL‐6 is a well‐established pro‐inflammatory cytokine with detrimental effects on skeletal muscle homoeostasis. Increased circulating levels of IL‐6 have been shown to disrupt the redox balance in skeletal muscle, leading to oxidative stress and impaired muscle function, as demonstrated by Forcina et al. in animal models [62]. Consistently, data from our group indicate that women with obesity exhibit significantly elevated levels of oxidative stress markers compared with normal‐weight women, with strong correlations to BMI, and visceral fat [63]. The observed reduction in IL‐6 gene expression in the FEHC group suggests that this dietary intervention offers a protective effect against IL‐6‐mediated inflammation, promoting the maintenance of redox balance and muscle integrity. This protective effect aligns with findings from other studies, which highlight the ability of dietary interventions to modulate IL‐6 levels and mitigate its adverse effects on muscle tissue. For instance, a short‐term high‐fat diet has been shown to lower glutathione levels, an important antioxidant, and increase IL‐6 expression in the skeletal muscle of animal models, emphasising the role of diet‐induced changes in IL‐6 in influencing muscle oxidative stress and function [64]. Furthermore, research demonstrates that exercise‐induced IL‐6 release from skeletal muscle can impact systemic inflammation and muscle metabolism, underscoring the complex role of IL‐6 in muscle physiology [65]. Collectively, these findings support the anti‐inflammatory potential of the FEHC diet in reducing IL‐6 levels, contributing to the preservation of muscle health and function. Notably, IL‐6 does not only promote oxidative stress but also interferes with anabolic signalling in muscle tissue. In vitro studies have demonstrated that IL‐6 overexpression suppresses the expression of IGF‐1, a key growth factor involved in muscle maintenance, regeneration, and protein synthesis [66, 67]. In this context, the reduction in IL‐6 gene expression observed in the FEHC group may represent a mechanism through which the diet exerts a protective effect on muscle homoeostasis, possibly supporting a more favourable anabolic environment. In line with this, our data revealed a trend towards increased IGF‐1 gene expression in the muscle tissue of the FEHC group. Supporting this interpretation, a recent study showed that skeletal muscle of individuals with obesity, who often exhibit chronically elevated IL‐6 levels, also displays lower protein synthesis rates, in parallel with reduced expression of muscle IGF‐1, IGF‐1 receptor, and specific IGF‐1 mRNA splice variants [68]. These observations reinforce the hypothesis that IL‐6 may act as a key upstream regulator linking metabolic inflammation to impaired muscle anabolism. Beyond its effects on bone tissue, the FEHC diet may also influence muscle regeneration through the non‐canonical WNT signalling pathway. Wnt5a, a key ligand in this pathway, has been shown to play a critical role in skeletal muscle development and regeneration. Research highlights that Wnt5a is upregulated during the early phases of muscle repair, suggesting its involvement in the activation and proliferation of muscle stem cells [69]. Our study revealed a trend of increased WNT5A expression in the muscle tissue of the FEHC group, pointing to the potential of this pathway in promoting muscle repair and regeneration. This observation is consistent with findings from Wang et al., who demonstrated that Wnt5a supports myogenic differentiation and muscle regeneration by regulating the expression of key myogenic factors, including MyoD, Myf5 and Myogenin, while facilitating intracellular calcium signalling through the opening of Ca^2+^ channels [70]. Collectively, these findings emphasise the potential of dietary interventions such as the FEHC diet to modulate essential molecular pathways, such as Wnt5a signalling, contributing to musculoskeletal health and improved muscle regeneration. Our investigation highlights several strengths while acknowledging certain limitations. A key strength of this study is the integration of clinical, systemic, and molecular assessments, allowing for a comprehensive understanding of the effects of the FEHC diet. Importantly, the study included a cohort of older adults with obesity, a high‐risk population for frailty and musculoskeletal health decline, enhancing the real‐world relevance of the findings. A particularly distinctive strength is the use of advanced gene expression analysis directly on human muscle and bone tissue. This innovative approach provides important insights into the molecular mechanisms underpinning the clinical effects observed. These analyses represent a significant advancement in understanding how dietary interventions can influence inflammation at a tissue‐specific level. Nonetheless, certain limitations should be acknowledged. The relatively modest sample size limits the statistical power to detect more subtle molecular effects, while the short trial duration restricts the ability to assess long‐term outcomes, such as the sustainability of benefits to musculoskeletal health. Moreover, although the FEHC group statistically significant waist circumference reductions, the targeted 10% weight loss was not achieved. This may have attenuated the magnitude of clinical and molecular effects typically associated with a pronounced weight reduction. In addition, a limitation concerns the timing of DXA measurements, which were performed post‐operatively, after the FEHC diet had been modified to include fish‐derived proteins. Therefore, DXA outcomes reflect the combined effects of both the strictly plant‐based pre‐surgical phase and the less restrictive post‐surgical phase. Additionally, insulin resistance was not assessed, as fasting insulin levels were not measured. This precluded the calculation of HOMA‐IR or similar indices, and fasting glucose alone was insufficient to characterise metabolic status. Including a direct measure of insulin sensitivity would have provided valuable insights into the metabolic impact of the intervention on skeletal muscle [71]. Finally, gene expression analyses in bone and muscle tissues were conducted at a single post‐intervention time point. As such, these data offer only cross‐sectional insight and do not allow for inference of longitudinal or causal effects of the dietary intervention. Furthermore, while the FEHC diet has shown promising results, further studies are needed to confirm its applicability across broader populations.

## 5 Conclusion

FEHC is effective in reducing systemic and local inflammation without affecting skeletal health. Future studies could build on these findings to further clarify the role of dietary interventions in preventing frailty in older adults with obesity.

## Author Contributions

F.C. contributed to investigation, patient management, and data curation. V.V. contributed to conceptualization, investigation, writing – original draft, and writing – review and editing. G.L. contributed to investigation, writing – original draft, and writing – review and editing. A.L. was responsible for formal analysis, data visualization, and statistical methodology. M.F., F.T. and A.P. contributed to the investigation and laboratory analyses. R.S. contributed to the methodology, experimental design and data acquisition. M.M. contributed to supervision, methodology, and protocol optimisation. F.R., G.V. and R.P. were involved in resources (surgical sample collection) and investigation. V.S., G.L., G.B. and C.V. contributed to serum sample processing, laboratory analyses, and data curation. N.N. was responsible for conceptualization, project administration, funding acquisition and writing, review and editing. All authors reviewed and approved the final version of the manuscript.

## Conflicts of Interest

The authors declare no conflicts of interest.

## Peer Review

The peer review history for this article is available at https://www.webofscience.com/api/gateway/wos/peer-review/10.1002/dmrr.70089.

## Supporting information


**Table S1**: Bone microarchitectural parameters assessed by μCT at T2. Data are showed as mean ± standard deviation.

## Data Availability

The data that support the findings of this study are available from the corresponding author upon reasonable request.
