# Supplementary material for: Fibre‐Enriched High‐Carbohydrate (FEHC) Diet Modulates Inflammation Without Affecting Bone Health in Older Women With Obesity: A Randomised Clinical Trial
Source: Diabetes Metab Res Rev. 2025 Sep 30;41(7):e70089. doi: 10.1002/dmrr.70089 (PMC12483497; doi:10.1002/dmrr.70089)
Supplement: Supplementary file 1 — Table S1: Bone microarchitectural parameters assessed by μCT at T2. Data are showed as mean ± standard deviation. [file DMRR-41-e70089-s001.docx]

**Fiber-Enriched High-Carbohydrate (FEHC) Diet Modulates Inflammation Without Affecting Bone Health in Older Women With Obesity: a Randomized Clinical Trial**

Francesca Cannata, Viola Viola, Giulia Leanza et al

**ONLINE SUPPLEMENTARY MATERIAL**

**Supplementary Table 1**

| **Parameters** | **ALD**  **(n=25)** | **FEHC**  **(n=32)** | **CD**  **(n=29)** | **P VALUE** |
| --- | --- | --- | --- | --- |
| **Total vBMD (mg/cm³)** | 264.16 ± 58.06 | 284.66 ± 47.52 | 265.19 ± 62.22 | ns |
| **Cross-sectional Area (mm²)** | 385.21 ± 76.33 | 396.44 ± 56.23 | 394.41 ± 65.12 | ns |
| **Trabecular vBMD (mg/cm³)** | 175.94 ± 42.18 | 169.20 ± 46.34 | 165.50 ± 38.40 | ns |
| **Trabecular area (mm²)** | 342.82 ± 68.93 | 345.16 ± 66.86 | 349.02 ± 68.19 | ns |
| **Trabecular number (mm⁻¹)** | 1.44 ± 0.26 | 1.41 ± 0.24 | 1.45 ± 0.22 | ns |
| **Trabecular thickness (mm)** | 0.27 ± 0.02 | 0.28 ± 0.02 | 0.26 ± 0.01 | ns |
| **Cortical vBMD (mg/cm³)** | 785.63 ± 69.14 | 764.32 ± 68.16 | 778.59 ± 63.10 | ns |
| **Cortical area (mm²)** | 65.02 ± 12.12 | 63.94 ± 12.80 | 65.01 ± 12.33 | ns |
| **Cortical thickness (mm)** | 0.94 ± 0.22 | 0.95 ± 0.33 | 0.86 ± 0.33 | ns |
| **Cortical porosity (%)** | 0.025 ± 0.01 | 0.030 ± 0.01 | 0.028 ± 0.01 | ns |

**Supplementary Table 1**. Bone microarchitectural parameters assessed by μCT at T2. Data are showed as mean ± standard deviation.
